# Case Report: Low-Level Maternal Mosaicism of a Novel CREBBP Variant Causes Recurrent Rubinstein-Taybi Syndrome in Two Siblings of a Chinese Family

**DOI:** 10.3389/fgene.2021.640992

**Published:** 2021-03-04

**Authors:** Shaobin Lin, Zhiming He, Linhuan Huang, Jialiu Liu, Ting Lei, Jianzhu Wu, Peizhi Huang, Yi Zhou, Yanmin Luo

**Affiliations:** ^1^Department of Obstetrics and Gynecology, The First Affiliated Hospital of Sun Yat-sen University, Guangzhou, China; ^2^Department of Ultrasonic Medicine, The First Affiliated Hospital of Sun Yat-sen University, Guangzhou, China

**Keywords:** CREBBP, germline mosaicism, Rubinstein-Taybi syndrome, next-generation sequencing, RNA sequencing

## Abstract

Familial Rubinstein-Taybi syndrome (RSTS) with recurrent RSTS siblings and apparently unaffected parents is rare; such cases might result from parental somatic and/or germline mosaicism. Parental low-level (<10%) germline mosaicism in the CREBBP-associated RSTS family has not been reported. Here, we present our studies of a Chinese family with two RSTS siblings and apparently unaffected parents. We detected the apparent de novo variant (DNV) c.3235C>T (p.Gln1079^*^) in CREBBP in the siblings via trio whole-exome sequencing. High-depth next-generation sequencing (NGS) for the parents revealed a low-level (<10%) mosaic variant in both the peripheral blood (3.64%) and buccal mucosa (1.94%) of the unaffected mother, indicating maternal somatic and germline mosaicism. Peripheral blood RNA-sequencing analysis for the patients and normal individuals indicated that the c.3235C>T (p.Gln1079^*^) non-sense variant did not trigger nonsense-mediated mRNA decay to reduce CREBBP mRNA levels. Transcriptome analysis revealed 151 downregulated mRNAs and 132 upregulated mRNAs between the patients and normal individuals. This study emphasizes that high-depth NGS using multiple specimens might be applied for a family with an affected sibling caused by an apparent CREBBP DNV to identify potential low-level parental mosaicism and provide an assessment of recurrence risk.

## Introduction

Rubinstein-Taybi syndrome (RSTS) is a rare genetic syndrome featuring distinctive facial dysmorphisms, characteristic hand and foot findings, short stature, developmental delay, and intellectual disability, with a reported incidence of 1:100,000–1:125,000 births in the Netherlands (Hennekam et al., 1990; Wiley et al., 2003; Hennekam, 2006). CREBBP gene variants, including single-nucleotide variants and exonic or whole-gene deletions, are reported to be the cause of RSTS type 1 (RSTS1; OMIM #180849), with autosomal dominant inheritance, contributing to ~50–60% of RSTS cases (Korzus, 2017). In families with clinically unaffected parents, CREBBP variants are considered to mostly derive from a de novo event (Bartsch et al., 2010). Although the recurrence risk in RSTS families is generally regarded to be low, either an unrecognized mild phenotype in a parent with heterozygosity (or somatic mosaicism) or unidentified germline mosaicism in an unaffected parent is presumed to contribute to a risk of RSTS recurrence in their offspring. The recurrence risk of a family with an affected sibling caused by an apparently de novo variant (DNV) is estimated to range from 0.048 to 9.4% (Campbell et al., 2014).

Overall, the detection of low-level mosaicism where the percent of cells with the variant are <10% with variant allele fractions (VAFs) <10% is challenging, while the detection of high-level mosaicism where the percent of cells with the variant are more than 10% with VAFs ≥10% is currently expected to be feasible (Gambin et al., 2020; Rodríguez-Martín et al., 2020). Nevertheless, recent advances in high-sensitivity genetic tests, including high-depth next-generation sequencing (NGS) and droplet digital PCR (ddPCR), have allowed for the more precise detection of low-level mosaicism that might be missed by traditional Sanger sequencing or low-depth NGS (Cao et al., 2019; Hu et al., 2019). In addition, it has been reported that routine exome sequencing (ES) variant calling pipelines are unable to detect variants with VAFs lower than 10% (Gambin et al., 2020). Therefore, it might be questioned whether some of the previously reported, presumed DNVs in probands might derive from parents with unidentified low-level somatic and/or germline mosaicism (Jonsson et al., 2018; Hu et al., 2019; Gambin et al., 2020). Here, we present our studies of a Chinese family with two boys with RSTS and apparently unaffected parents. With the use of trio whole-exome sequencing (WES), we detected a novel CREBBP variant in the two children; subsequent high-depth NGS identified a mosaic variant in the mother, indicating low-level maternal germline mosaicism (<10%) for the first time.

## Case Presentation

**Figure 1:**
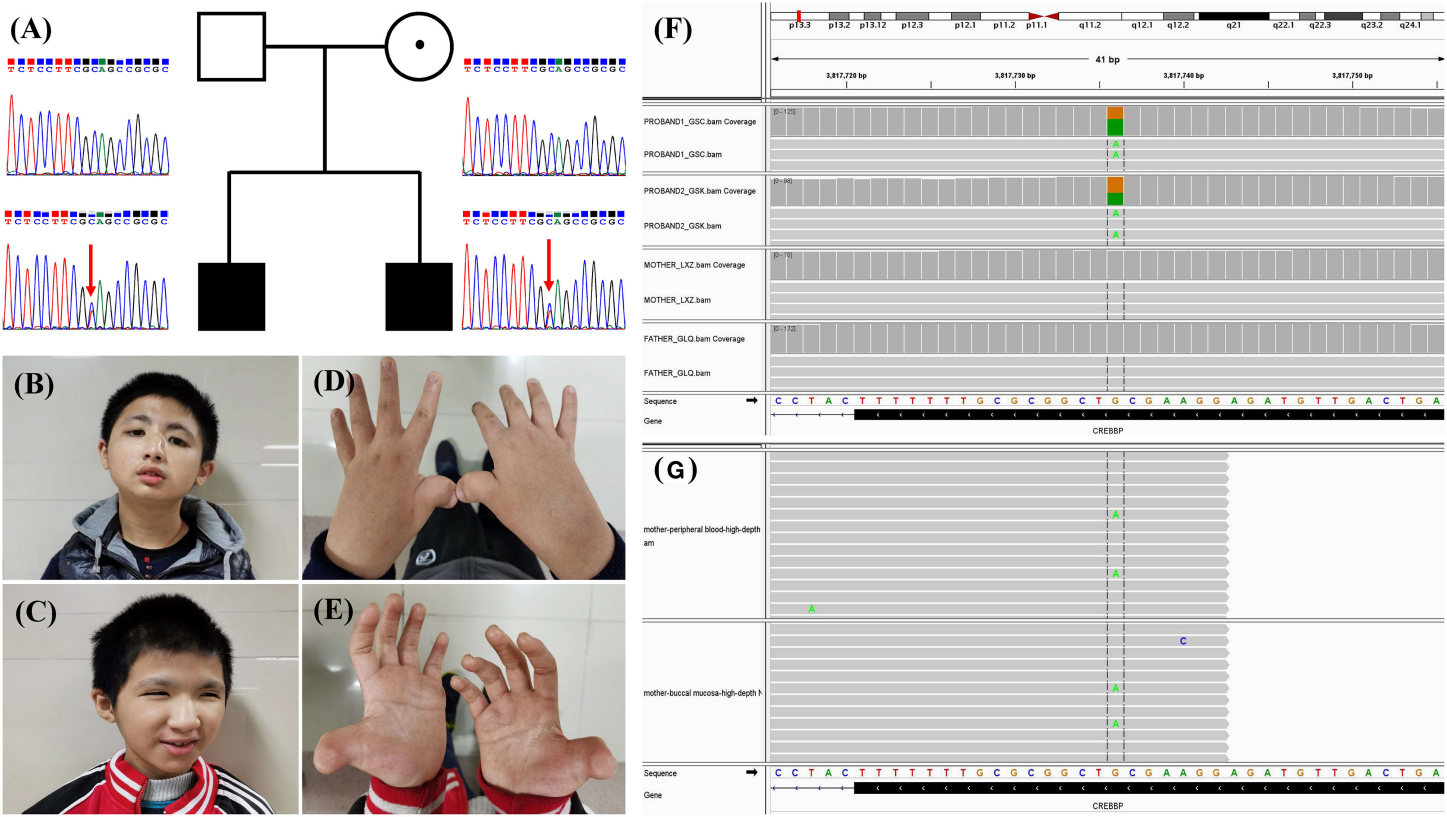
**(A)** Family pedigree and Sanger sequencing results of the CREBBP variant. The arrows indicate the variant c.3235C>T in the siblings. **(B,C)** Photos of the two siblings. **(D,E)** Hand morphology of the two siblings. Typical features include broad and angulated thumbs. **(F)** Sequence read alignment of the c.3235C>T (16:3817736:G>A) site from the BAM files of trio whole-exome sequencing using Integrative Genomics Viewer (v2.8.1) indicated a variant allele fraction for c.3235C>T of ~50% in the siblings but not in the parents. **(G)** Sequence read alignment of the c.3235C>T (16:3817736:G>A) site from the BAM files of high-depth next-generation sequencing using Integrative Genomics Viewer (v2.8.1) indicated a variant allele fraction for c.3235C>T of ~3.64% (336/9225 reads) and 1.94% (173/8896 reads) in the peripheral blood and buccal mucosa of the mother, respectively.

Two male children presented to our genetics clinic at the ages of 13 and 16 years. They were born to a non-consanguineous family, and abnormal pregnancy or delivery events were not noted. According to their medical records and descriptions, the parents had no recognizable phenotype or significant medical conditions. At the time that the two children were referred for clinical genetic assessment, the height of the older and younger brother was 152 cm (<3rd percentile) and 130 cm (<3rd percentile), and their weights were 53 kg (25th percentile) and 40 kg (19th percentile), respectively. The boys were able to walk alone at the ages 25 and 28 months and spoke their first words at 23 and 25 months, respectively. They showed common clinical features, including the characteristic facial appearance, short stature, speech and motor delay, intellectual disability, microcephaly, hearing impairment, glaucoma, crowding of teeth and malocclusion, broad and angulated thumbs and halluces, bilateral syndactyly between the great and second toe, and cryptorchidism (Figure 1). The characteristic facial appearance includes marked eyebrows, downslanted palpebral fissures, broad and prominent nose, low-hanging columella, strabismus, and micrognathia (Figure 1). Behavior problems were observed, with aggressive behaviors, distractibility, instability of mood, and stereotypies. Other medical conditions included recurrent upper respiratory infections, gastroesophageal reflux, and constipation. The parents provided informed consent for this research and publication of the data.

## Methods

### Karyotyping and Chromosomal Microarray Analysis

Traditional G-banded karyotyping was performed on peripheral blood using standard protocols. CMA was conducted using a single-nucleotide polymorphism (SNP) array platform with Affymetrix CytoScan HD arrays (Thermo Fisher Scientific, Inc., Waltham, Massachusetts, USA) according to the manufacturer's protocols.

### WES

Exome sequences were captured by NimbleGen SeqCap EZ Exome v3 (Roche NimbleGen, Madison, Wisconsin, USA); after enrichment and purification, the exome libraries were sequenced using the NextSeq500 platform (Illumina, San Diego, CA, USA). Sequence reads were aligned to the February 2009 human reference sequence (GRCh37/hg19) assembly. The sequencing process, variant filtration and interpretation followed the protocols in a previous study (Fu et al., 2020).

### Sanger Sequencing

PCR and Sanger sequencing were performed using standard methods. The primer sequences used for PCR and sequencing are available upon request.

### PCR Amplicon-Based High-Depth NGS

PCR amplicon-based NGS was performed as previously described (Gambin et al., 2020). Briefly, parental genomic DNA from peripheral blood, sperm, and buccal mucosa was amplified using primers targeting putative mosaic variants. The PCR products were purified, and the purified amplicons were sequenced using the Illumina NovaSeq 6000 platform (Illumina, San Diego, CA, USA).

### RNA Sequencing

Total RNA was extracted from peripheral blood using TRIzol (Thermo Fisher Scientific, Inc., Waltham, Massachusetts, USA). RNA-Seq libraries were prepared using 3 μg of total RNA. An Epicenter Ribo-Zero™ rRNA Removal Kit (Epicenter, USA) was employed to remove ribosomal RNA and a Directional RNA Library Prep Kit (NEB, USA) to prepare the sequencing libraries. The Illumina HiSeq 4000 (Illumina, San Diego, CA, USA) was utilized for RNA sequencing. The RNA sequencing procedure, data processing and analysis were in accordance with a previous study (Fu et al., 2020).

## Results

### Karyotyping and CMA

G-banded karyotyping and CMA did not reveal relevant chromosomal abnormalities, copy number variants (>100 kb) or runs of homozygosity (>5 Mb) in the two siblings.

### WES, Sanger Sequencing, and Amplicon-Based High-Depth NGS

The mean coverage depth for the WES data was ≥160×, with more than 99.8% of the target regions covered at ≥20×. Through a gene filtration and bioinformatic analysis process, a de novo nonsense variant 16:3817736:G>A, c.3235C>T (p.Gln1079^*^), in exon 16 of CREBBP (NM_004380.3) was identified in the two children (Figure 1). The variant is absent in public population genomic databases. The variant is classified as likely pathogenic according to American College of Medical Genetics (ACMG) variant interpretation guidelines.

Intriguingly, WES did not reveal the variant in the unaffected parents (Figure 1). Moreover, Sanger sequencing confirmed the heterozygous c.3235C>T (p.Q1079^*^) variant in the two affected children but not in the parents (Figure 1). We then employed PCR amplicon-based high-depth NGS to investigate a putative mosaic variant in one of the parents and ultimately identified mosaic c.3235C>T (p.Q1079^*^) variants in the peripheral blood (3.64%, alternate reads/total reads = 336/9225) and buccal mucosa (1.94%, alternate reads/total reads = 173/8896) of the mother but not the father (Figure 1).

### RNA Sequencing

We then investigated whether the c.3235C>T variant influences CREBBP mRNA levels using RNA-sequencing analysis of peripheral blood from the patients and normal individuals. However, the level of CREBBP mRNA was not significantly different between the patients and normal individuals [log2 ratio (patients/controls) = −0.0032274829851164; *Q*-value = 0.996318464271172].

**Figure 2:**
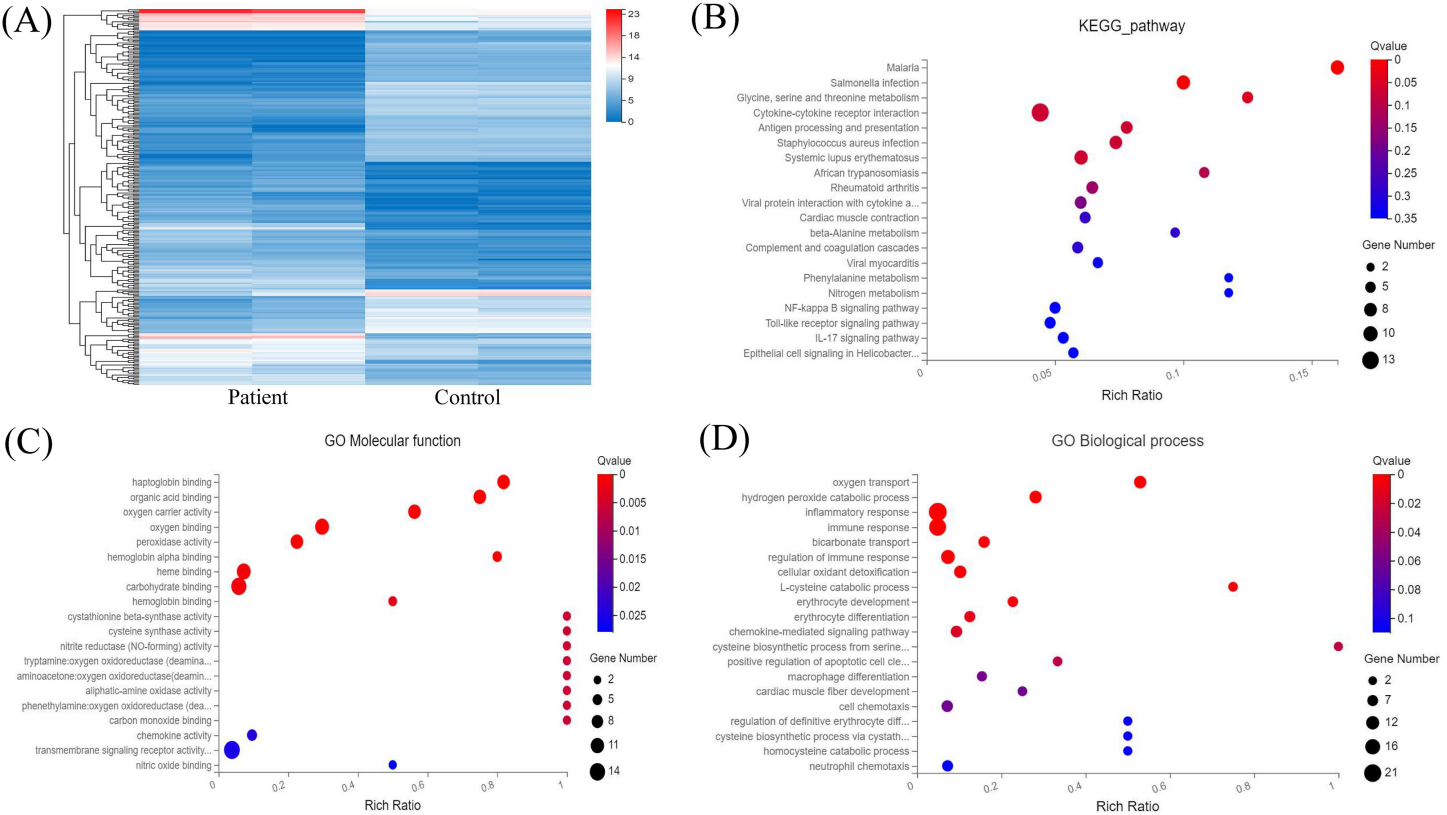
RNA sequencing analysis of the peripheral blood from the patients and normal individuals and functional annotation of mRNAs. **(A)** Heat map of mRNAs with differential expression in peripheral blood from the patients and normal individuals. **(B)** KEGG pathway analysis of mRNAs with differential expression. **(C,D)** GO enrichment analysis of mRNAs with differential expression.

Transcriptome analysis was then applied to compare peripheral blood genome-wide expression profiles between the patients and normal individuals. In total, 283 genes showed significantly differential expression between them. Among these, 151 mRNAs were downregulated, and another 132 mRNAs were upregulated. The top 20 genes with significantly differential mRNA expression are listed in Table 1, of which 10 were upregulated and the other 10 downregulated. Furthermore, we performed Gene Ontology (GO) and Kyoto Encyclopedia of Genes and Genomes (KEGG) analyses for all differentially expressed mRNAs induced by the CREBBP c.3235C>T variant. GO analysis revealed these mRNAs to be mainly enriched in transmembrane signaling receptor activity and inflammatory response terms. Based on the KEGG analysis, these mRNAs are mostly associated with pathway in the cytokine-cytokine receptor interaction (Figure 2).

**Table 1:** Top 10 upregulated and 10 downregulated mRNAs between the patients and normal individuals.

**Gene symbol**	**Patients expression**	**Normal individuals expression**	**Patients average read count**	**Normal individuals average read count**	**log2 ratio**	**Up/down regulation**	***P*-value**	***Q*-value**
HLA-DRB5	225.835	0.765	3532.39	13.875	7.482495967	up	4.91E-55	1.78E-52
MYBL1	26.42	0.65	1820	51.5	4.544114327	up	8.29E-42	2.47E-39
MYOM2	38.7	1.05	2678.5	78.5	4.529897009	up	1.13E-35	2.32E-33
FOSB	19.76	0.52	1006	29.5	4.506423885	up	2.03E-09	3.04E-08
KLRF1	68.75	2.09	1035	36.5	4.259903293	up	5.50E-25	6.66E-23
CXCL8	42.075	1.37	890	32.5	4.218315485	up	1.28E-11	3.02E-10
G0S2	50.5	1.29	574	20	4.161368474	up	1.36E-08	1.63E-07
RGS1	11.715	0.375	207	8	4.086850386	up	6.25E-10	1.05E-08
RPL36A	262.95	54.965	1548.5	422	4.083172057	up	1.11E-12	3.43E-11
RPL34	1522.475	59.685	9236	422	3.965364098	up	1.23E-11	2.91E-10
ALAS2	1.745	878.28	45.5	26335.5	−9.768604841	down	1.24E-212	4.07E-209
SLC4A1	0.455	82.065	22.5	6477	−8.813880937	down	4.48E-90	2.93E-87
IFI27	0.94	68.11	6	535	−7.096128608	down	2.88E-38	7.25E-36
SLC6A8	0.545	16.94	23.5	984.5	−6.056603232	down	4.39E-37	1.02E-34
PHOSPHO1	6.32	128.8	171	4585	−5.432012286	down	2.61E-17	1.53E-15
SLC25A39	44.395	819.91	1020.5	21348	−5.05753814	down	3.05E-36	6.64E-34
PDZK1IP1	1.49	19.975	15.5	291	−4.951911648	down	5.28E-11	1.11E-09
NLRP6	0.835	13.215	32.5	565.5	−4.679911744	down	1.89E-27	2.95E-25
MMP9	3.01	31.465	93	1361.5	−4.630504037	down	0.006132	0.009425
ALPL	5.28	64.135	182.5	2705.5	−4.580305046	down	9.21E-14	3.34E-12

## Discussion

To date, the majority of CREBBP-associated RSTS are sporadic cases caused by DNVs. Familial CREBBP-associated RSTS in a parent-to-child transmission manner or a recurrent condition with multiple affected siblings and apparently unaffected parents in a family might result from parental somatic and/or germline mosaicism. Indeed, CREBBP mosaic variants have been previously reported in eight RSTS cases (Table 2) (Gervasini et al., 2007; Chiang et al., 2009; Bartsch et al., 2010; Tajir et al., 2013; De Vries et al., 2016). In the study from Gervasini et al. (2007), high-level (ranging from 17 to 30%) somatic mosaicisms of CREBBP partial deletions were identified via fluorescence *in situ* hybridization in three cases with RSTS. De Vries et al. (2016) also reported a somatic mosaicism of CREBBP c.5039_5041delCCT (p.Ser1680del) in the buccal mucosa but not the peripheral blood from a patient with RSTS and Filippi syndrome. However, whether germline mosaicisms were also present in these cases in the two studies remained unknown. To our knowledge, parental germline mosaicism of CREBBP was assumed in four families (Chiang et al., 2009; Bartsch et al., 2010; Tajir et al., 2013). All of them showed high-level mosaic variants and were expected to be easily detected by Sanger sequencing or pyrosequencing. However, we noted that there was sufficient evidence supporting an assumed parental germline mosaicism in the family reported by Bartsch et al. (2010), in which the mosaic c.4134G>T (p.Arg>Arg) was detected by Sanger sequencing or pyrosequencing in the peripheral blood and buccal mucosa from the proband's father. Additionally, there was no sufficient evidence supporting a confirmation of parental germline mosaicism in the three families reported by Chiang et al. (2009) and Tajir et al. (2013), in which the mosaic CREBBP variants were not revealed by Sanger sequencing in the peripheral blood and buccal mucosa from the proband's parents. Of note, low-level parental germline mosaicism could still not be excluded by Sanger sequencing or pyrosequencing without high-sensitivity performance on the detection of mosaicism. Actually, many reports do not provide direct evidence for germline mosaicism because direct identification of germline mosaicism for females is difficult (possibly invasive ovarian tissue collection), though it is feasible for males (sperm).

**Table 2:** Summary of previously reported cases with mosaic variants in CREBBP.

**Study**	**Case**	**CREBBP variant in the proband**	**CREBBP variant in one of the parents**	**Mosaic ratio**	**Detection method**	**Somatic mosaicism**	**Germline mosaicism**	**Clinical findings in cases with (assumed) mosaicism**	**Familial or recurrent RSTS**
Gervasini et al. (2007)	case 38	mos deletion	unk	peripheral blood (30%) buccal mucosa (unk)	FISH	+	unk	RSTS	unk
	case 40	mos deletion	unk	peripheral blood (20%) buccal mucosa (17%)	FISH	+	unk	RSTS	unk
	case 66	mos deletion	unk	peripheral blood (22%) buccal mucosa (21%)	FISH	+	unk	RSTS	unk
Chiang et al. (2009)	family 1- one of the parents	6122-6125delCCAT	-	peripheral blood (0) buccal mucosa (0)	Sanger	–	assumed	broad great toes in the father	+
	family 2-father	c.4078C>T (p.Arg1360*)	mos c.4078C>T (p.Arg1360*)	peripheral blood (ND) buccal mucosa (ND)	Sanger	ND	assumed	broad great toes	–
Bartsch et al. (2010)	family 2-father	c.4134G>T (p.Arg>Arg)	mos c.4134G>T (p.Arg>Arg)	peripheral blood (38%) buccal mucosa (31%)	Sanger and Pyrosequencing	+	assumed	broad thumbs and great toes	+
Tajir et al. (2013)	one of the parents	c.4361T>A (p.Leu1454His)	-	peripheral blood (0) buccal mucosa (0)	Sanger	–	assumed	-	+
De Vries et al. (2016)	one of the parents	mos c.5039_5041delCCT (p.Ser1680del)	-	peripheral blood (0) buccal mucosa (ND)	Sanger, RELP and WES	+	unk	RSTS and Filippi syndrome	–
Current study	mother	c.3235C>T (p.Gln1079*)	mos c.3235C>T (p.Gln1079*)	peripheral blood (3.64%) buccal mucosa (1.94%)	high-depth NGS	+	assumed	RSTS	+

*mos, mosaic; unk, unknown; ND, not determined; FISH, fluorescence in situ hybridization; RELP, restriction fragment length polymorphism analysis; WES, whole-exome sequencing; NGS, next-generation sequencing*.

Regardless, high-depth NGS and ddPCR have enabled assessing somatic and/or germline mosaicism in families with DNVs (Hu et al., 2019). Here, we report a novel heterozygous CREBBP variant in two siblings with unique clinical features in a Chinese family. The phenotypes of the two siblings are highly consistent with classical RSTS. Because routine trio-WES analysis did not identify the variant in the unaffected parents, parental low-level germline mosaicism was suspected and then confirmed by high-depth NGS. In this study, we used multiple specimens, including peripheral blood, sperm and buccal mucosa, to investigate c.3235C>T (p.Gln1079^*^) mosaicism in the parents. Consequently, the mother was revealed to be a carrier of a c.3235C>T (p.Gln1079^*^) low-level mosaic variant in the peripheral blood (3.64%) and buccal mucosa (1.94%), indicating maternal somatic and germline mosaicism.

To date, 209 unique pathogenic or likely pathogenic variants of CREBBP have been recorded in the Leiden Open Variation Database (http://www.LOVD.nl/CREBBP). Approximately 50% of pathogenic variants related to CREBBP-associated RSTS cluster in chromatin-modifying regions (HAT and Bromo domains) in the CREBBP gene, especially the former (Korzus, 2017). A previous study demonstrated that the sole loss of HAT activity was sufficient to cause RSTS (Kalkhoven et al., 2003). The c.3235C>T (p. Gln1079^*^) variant is located in exon 16 and maps to the region between the KIX and Bromo domains. Very few variants in exon 16 are reported to contribute to RSTS (Korzus, 2017). In fact, only one, c.3096dup (p.Lys1033^*^), is located in exon 16 according to the Leiden Open Variation Database. The c.3235C>T (p.Gln1079^*^) variant is predicted to produce a truncated protein consisting of 1079 amino acids excluding the Bromo, HAT, and TAZ2 domains. Loss of these critical domains might affect the wild-type protein and might lead to haploinsufficiency to cause RSTS, which is also supported by the observation that the two siblings had consistent phenotypes matching classic RSTS.

CREBBP transcription produces an ~10 kb mRNA containing a 7.3 kb coding sequence. CREBBP is extensively expressed in various tissues in humans, including peripheral blood mononuclear cells. RNA sequencing of peripheral blood from the patients and normal individuals demonstrated no difference in mRNA levels between them. Thus, c.3235C>T (p.Gln1079^*^) mutant transcripts might be stable, with transcription levels similar to those of the wild-type transcripts, and the c.3235C>T (p.Gln1079^*^) variant in exon 16 of CREBBP might escape nonsense-mediated mRNA decay. We also established peripheral blood mRNA profiles for the patients and normal individuals and detected 151 downregulated and 132 upregulated mRNAs between them. GO and KEGG pathway analyses revealed that these mRNAs are enriched in transmembrane signaling receptor activity and inflammatory response terms and pathway in the cytokine-cytokine receptor interaction. Overall, further research should be performed to investigate whether the c.3235C>T (p.Gln1079^*^) variant contributes to RSTS via these pathways.

Parental somatic and/or germline mosaicism causing recurrent autosomal dominant or X-linked genetic disorders in a family are rare but not uncommon (Smith et al., 1999; Aarabi et al., 2018; Hu et al., 2019; Liu et al., 2019). To our knowledge, parental low-level (<10%) somatic and germline mosaicism in a CREBBP-associated RSTS family has not been reported with sufficient evidence. Together with many previous studies and given the recurrent probability of a family with an affected sibling caused by an apparent DNV in CREBBP, we suggest that further high-depth NGS detection in multiple specimens might be offered to identify potential low-level parental mosaicism in an effort to provide an assessment of recurrence risk in these families.

## Data Availability Statement

The supporting data for the current study are available from the corresponding authors upon reasonable request.

## Ethics Statement

The studies involving human participants were reviewed and approved by Ethics Committee of The First Affiliated Hospital of Sun Yat-sen University. Written informed consent to participate in this study was provided by the participants' legal guardian/next of kin. Written informed consent was obtained from the individual(s), and minor(s)' legal guardian/next of kin, for the publication of any potentially identifiable images or data included in this article.

## Author Contributions

SL, YZ, and YL designed the study and wrote the manuscript. ZH, LH, and TL collected the clinical data and samples from the family. JL, JW, and PH performed the genetic analysis and evaluation of the variants. All authors contributed to the article and approved the submission.

## Conflict of Interest

The authors declare that the research was conducted in the absence of any commercial or financial relationships that could be construed as a potential conflict of interest.
